# Animal Chemistry

**Published:** 1838-07

**Authors:** 


### ANIMAL CHEMISTRY.

### *Microscopical Researches on the Composition of the Vaccine Fluid.* By M. Dubois, of Amiens.

[This is a memoir read before the Royal Academy of Medicine: we can only find room for the concluding part of it, which gives the summary of the results obtained.]

1st. The vaccine virus, whether liquid or dry, shows no signs of globules.

2d. The same virus, examined by means of the strongest magnifiers, shows no traces of animalcules.

3d. In its recent state, (that is to say, during the first hours that follow its removal from the pustule,) this virus is remarkably fluid and limpid: by degrees it takes a more solid form, and shows a kind of crystallization.

4th. In its state of desiccation, we observe two orders of physical arrangement: viz. 1, lengthened lines, both opaque and transparent, and very slightly interlacing; and, 2, a very minute network.

5th. These arrangements are essential to good vaccine lymph, and show themselves in all cases exactly in the same way.

6th. When these appearances of the virus are not to be found, the vaccine has lost its contagious properties.

7th. These material conditions may fail either from an anomalous development of the pustules, and consequently from a constitution previously vitiated, or from accidental causes.

8th. A high and low temperature (ebullition and congelation) hinder the establishment of these physical arrangements.

9th. When the vaccine virus, through the operation of these causes, has not been able to form itself in this way, it loses its contagious properties.

10th. It is not by killing the animalcules that a high and low temperature destroy the properties of vaccine, but by altering its material conditions.

11th. Microscopic examination of the fluid can assist in determining the existence or non-existence of its preservative properties.

*Bull. de l'Acad. Roy. de Med.* 30 *Avril,* 1838.

---

### *On the Comparative Analysis of Healthy Bile and that containing Calculi.* By Paulo Muratori.

The first experiments of the author were made upon biliary calculi, of which he found the white portions composed of pure cholesterine, the yellow of dried and altered bile.

In the experiments upon the bile, with or without calculi, he took care to use such re-agents as should not engender fresh products; as appears to have been the case in the analysis of Tiedemann and Gmelin.

A thousand parts of human bile are composed of

| | Healthy bile. | | With calculi. |
|---|---|---|---|
| Water | 832 | .. | 786 |
| Peculiar fatty matter | 5 | .. | 8 |
| Colouring matter | 11 | .. | 9 |
| Cholesterine with soda | 4; | without soda, | 35 |
| Picromel | 94.86 | .. | 96.5 |
| Fleshy extractive | 2.69 | .. | 5.25 |
| Mucus | 37 | .. | 46 |
| Soda | 5.14 | .. | 0 |
| Phosphate of soda | 3.45 | .. | 5.50 |
| Phosphate of zinc | 3 | .. | 5 |
| Chloride of sodium | 1.86 | .. | 3.75 |
| | 1000 | | 1000 |

From this analysis, and from other experiments, the author concludes that the cholesterine is not mechanically mixed with the bile, but that, combined with the soda, it forms a saponaceous compound, soluble in the bile. If, however, it be in excess, or the alkali be deficient, it is precipitated from the bile in a crystalline form. *Bullettino delle Sc. Med. de Bologna. Settemb.* 1836.

---

*Analysis of Liquor Amnii at two different Periods of Pregnancy.* By Dr. C. Vogt, of Bern.

| Liquor amnii, | at 3½ months; | at 6 months. |
|---|---|---|
| Water | 979.45 | 990.29 |
| Alcoholic extract, consisting of an uncertain animal matter and lactate of soda | 3,69 | 0.34 |
| Chloride of sodium | 5.95 | 2.40 |
| Albumen (as residuum) | 10.77 | 6.67 |
| By boiling (9.45). | | |
| Sulphate and phosphate of lime, and loss | 0.14 | 0.30 |
| | 1000.00 | 1000.00 |
| Specific gravity | 1.0182 | 1.0092 |

The first fluid was, in all its circumstances, more concentrated than the other. Whether this is a condition connected with the development of the fœtus, must be a subject of future investigation. The female, pregnant three months and a half, died of an inflammatory disease; the other in a state of cachexia. The fluids were drawn from the membranes by a canula, and thus obtained pure. This may explain the difference between the present analysis and that of Frommherz, who obtained the fluid as discharged in natural labour. *Müller's Archiv.*

---

*New Analysis of the Blood.* By M. Beudant.

The following is an abridgment of the conclusions drawn by M. B. from an examination of healthy blood:

1. That the albumen and gelatin are but the same substance, and that the albumen is only liquid in consequence of its combination with a mixture of thirteen parts of neutral salts, soluble in water, and one part of soda, contained in the blood.
2. The central corpuscles of the coloured globules of the blood are formed of solid albumen or fibrin.
3. In its healthy state the blood always contains some of the yellow matter of bile, which is also constantly met with, both in it and in the tissues of jaundiced persons.
4. The composition of the serum is always identical in healthy individuals.

The same is the case with the globules; and the different kinds of blood differ only in the relative proportions of these constituent parts.

5. The substances of which the serum and globules consist are in a very simple numerical proportion: thus, the serum being 1000, the salts are 10; the neutral fatty matters, together with the yellow and blue colouring matters, 20; the albumen, 80; and the whole of these solid substances, relatively to the water, which is 900, constitutes a total of 100.

*Archives génerales de Médecine. Janvier*, 1838.

*On the Presence of Urea in Dropsical Effusions.* By R. MARCHAND.

IN Poggendorff's Annalen, (vol. xxxviii. p. 356.) I communicated the analysis of a dropsical fluid, which was remarkable by containing urea, in the proportion of 0.42 per cent. Since that period I have twice had occasion to analyze dropsical fluids, and each time I have detected urea; in the first instance in the proportion of 0.68 per cent., and in the second in the proportion of 0.50 per cent. I have no doubt that the urea existed in these fluids in considerably larger quantity, but its separation becomes exceedingly difficult, owing to the accompanying albumen. I may remark, that in all the three cases there was retention of urine; and in two, which proved fatal, examination after death showed that peculiar degeneration of the kidney which has been described by Bright. It gives me much pleasure to see these analyses confirmed in a paper by Nysten, which, although read to the Academy of Sciences as early as 1810, did not appear in the Journal de Chimie Médicale till June, 1837. In this paper several cases are quoted, and amongst others one in which urea was detected in the perspiration, and another in which both urea and uric acid were found in the fluid ejected from the stomach. In how far do these facts favour the view that urea is prepared in the blood, and is not a product of the kidneys?

*Müller's Archiv. Jahrgang*, 1837. Heft iv.

---

